# Resistance to Extended-Spectrum β-Lactamases in *Salmonella* from a Broiler Supply Chain

**DOI:** 10.3390/ijerph111111718

**Published:** 2014-11-13

**Authors:** Jane Mary Lafayette Neves Gelinski, Amanda Bombassaro, César Milton Baratto, Vânia Aparecida Vicente

**Affiliations:** 1 Graduate Program in Science and Biotechnology, University of West of Santa Catarina—UNOESC, Videira 89.560-000, Brazil; E-Mail: cesar.baratto@unoesc.edu.br; 2 Graduate Program in Microbiology, Parasitology, and Immunology, Federal University of Paraná-UFPR, Curitiba 80.060-000, Brazil; E-Mails: amandabombassaro@hotmail.com (A.B.); vaniava63@gmail.com (V.A.V.)

**Keywords:** Enterobacteriaceae, environment, antimicrobials, bacterial pathogen

## Abstract

The prevalence of extended-spectrum β-lactamase (ESBL)-producing Enterobacteriaceae varies worldwide, however, the incidence of ESBL-producing environmental *Salmonella* isolates is increasing. *Salmonella* is still one of the most important pathogens that occur in the poultry supply chain. Therefore, this study analyzed the susceptibility of *Salmonella* isolates collected from a poultry supply chain to β-lactam antibiotics, and examined the phenotypes of the isolates based on enzyme-inducible AmpC β-lactamase analysis. All analysis of the putative positive isolates in the current study confirmed that 27.02% (77/285 analysis) of all ESBL tests realized with the isolates produced a profile of resistance consistent with β-lactamase production. All isolates of *S.* Minnesota serotype had ESBL phenotype. Aztreonam resistance was the least common amongst the *Salmonella* isolates, followed by ceftazidime. The presence of inducible chromosomal ESBL was detected in 14 different isolates of the 19 serotypes investigated. These results are very indicatives of the presence of ESBL genes in *Salmonella* isolates from a broiler supply chain, reaffirming the growing global problem of ESBL resistance.

## 1. Introduction

Data from the Food and Agriculture Organization and the United States Department of Agriculture shows that the largest chicken meat-producing countries are the United States of America and Brazil, with an estimated combined annual output for 2014 of over 40 million tons [1]. However, despite the huge amount of technology behind these numbers, the poultry sector is still facing new emergencies related to antimicrobial resistance of pathogens throughout the poultry production chain. 

Poultry is a major global reservoir of *Salmonella*; thus, control of this pathogen is important in the interests of public health. Related to this is the increasing number of enterobacterial strains producing extended-spectrum β-lactamases (ESBLs). These enzymes inactivate a wide variety of β-lactam drugs, including third-generation cephalosporins, penicillins, and monobactams [2,3,4]. An analysis of ESBL production in microorganisms not normally known to show β-lactam resistance can generate relevant information with respect to the transfer of resistance genes and the importance of control measures for the use of antibiotics in animal feed [5,6]. The prevalence of ESBL carriage is likely to increase and spread to different enteric pathogens, as occurred with ampicillin resistance [7] or more recently for cephalosporin resistance in *Escherichia coli*, even in the absence of selective pressure from antimicrobial agents [8]. 

In general, ESBL-producers are resistant to all penicillin, cephalosporin, and monolactam antibiotics [9]. ESBLs have evolved into families of enzymes encoded by plasmids (TEM, SHV, cefotaxime (CTXM), and oxacillin (OXA)), but depending on the bacterial species can also be encoded on the chromosome or be transposon-mediated [10,11]. This diversity has favored the spread of these enzymes, as in the case of TEM1, which hydrolyzes penicillins and first generation cephalosporins [12]. ESBLs have emerged and spread throughout the world in the few years since the introduction of new antibiotics that target β-lactamases, and are mainly produced by species of the family Enterobacteriaceae [4,5,13]. ESBL-producing Enterobacteriaceae have been isolated from all environments, suggesting a global expansion of these enzymes. 

The aim of this study was to investigate the presence of ESBLs in *Salmonella* isolates derived from the environmental poultry production chain. Susceptibility to β-lactam antimicrobials was examined, along with phenotypic analysis of the isolates based on the presence/absence of inducible AmpC β-lactamases.

## 2. Materials and Methods 

### 2.1. Salmonella Isolates

The samples were obtained from some farms (twenty-eight) belonging to one group of associated producers of broiler chickens (a poultry production chain) from Southern of Brazil (states of Paraná, Santa Catarina, and Rio Grande do Sul). The sampling was conducted twice a week for one month. The initial samples (750) were obtained from aviary environment (faeces and aviary bed) using swabs or spatulas for harvest in sterile plastic bags. The samples were processed for presence or absence of *Salmonella* spp. [14]. From these samples, two-hundred isolates were indicated as *Salmonella* spp. (140 of aviary bed and 60 of faeces). After analysis, nineteen different *Salmonella* serotypes were identified based on morphological, biochemical characteristics (API20E bioMérieux, São Paulo, Brazil), and serological tests (somatic antigens and flagella). All tests were performed according to standard methodology [15]. Serotyping of *Salmonella* isolates was performed by Adolfo Lutz Institute, São Paulo, Brazil.

### 2.2. Antimicrobial Susceptibility Testing and Phenotypic Screening for ESBL Production

**Figure 1:**
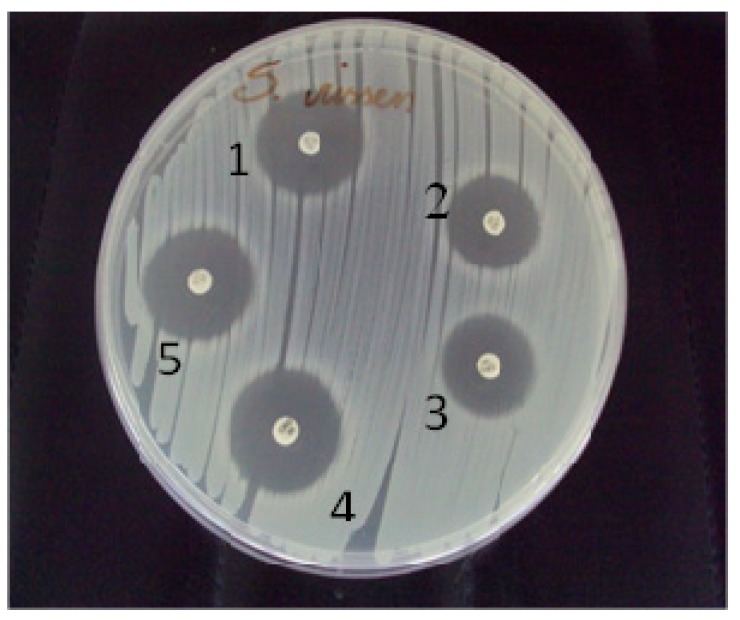
Initial screening for extended-spectrum beta-lactamase production by the disk diffusion method. Antibiotic disks (30 μg each): 1, aztreonam; 2, ceftazidime; 3, cefoxitin; 4, ceftriaxone, and 5, cefotaxime.

Nineteen different serotypes were identified amongst the 200 previously indicated as *Salmonella* spp. isolates. One representative isolate from each serotype was screened to evaluate β-lactamase production and resistance to major antimicrobial agents. The tests were carried out using the disk diffusion method and the guidelines of the National Committee for Clinical Laboratory Standards [6]. Briefly, a lawn of each isolate was prepared on Mueller-Hinton agar plates, and antibiotic-impregnated disks (Probac, São Paulo, Brazil) were placed on the plates. The following antibiotics were examined: aztreonam (30 μg), cefoxitin (30 μg), cefotaxime (30 μg), ceftazidime (30 μg), and ceftriaxone (30 μg) (Figure 1). Duplicate plates were prepared for each isolate and were incubated for 24 h at 35 °C. Sensitivity was evaluated by measuring the average diameters of inhibition zones formed around each of the disks. Isolates that showed sensitivity to any of the antimicrobials examined were considered to be possible ESBL-producers. The tests were performed in triplicate. A total of 285 analysis (isolates × 3 repetitions × 5 antibiotics) were performed. Breakpoints suggested by the CLSI [6] for the tested antibiotics were used to define the profile of ESBL production by the strains tested here. *Klebsiella pneumoniae* ATCC 700603 and *E. coli* ATCC 25922 were used as positive and negative controls, respectively.

### 2.3. Detection of Inducible Chromosomal β-Lactamases

The tests were performed on two consecutive days: a simple disk diffusion test was carried out on the first day, and a disk approximation test was carried out on the second day [6]. On the first day, the diameters of the inhibition zones of cefoxitin (inducer) and the β-lactam ceftazidime were recorded to calculate the distance at which the discs would be placed on the following day. Thus, a truncated area was apparent between the disks when the isolate produced β-lactamases. If the difference between the radius of the complete zone of inhibition and the radius of the truncated zone was ≥4 mm, the test was considered positive.

## 3. Results and Discussion

**Figure 2:**
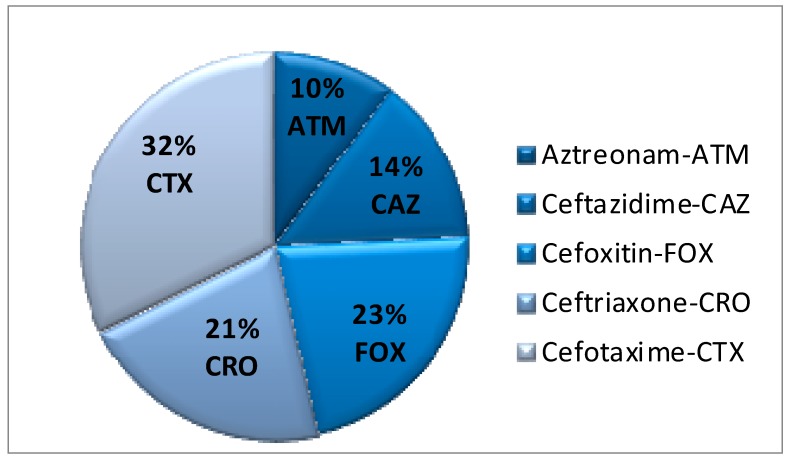
Percentage among the nineteen *Salmonella* serotypes isolates showing resistance to each of the tested β-lactam antibiotics.

Nineteen different *S. enterica* subspecie *enterica* serotypes were identified: *S*. Mandelia, *S.* Hadar, *S.* Enteritidis, *S*. Rissen, *S*. Tennessee, *S*. Cubana, *S*. Minnesota, *S*. Mbandaka, S. Agona, *S*. Albany, *S*. Virchow, *S*. Meleagridis, *S*. Anatum, *S*. Livingstone, *S*. Carrau, *S.* Schwarzengrund, *S*. Oranienburg, *S.* Worthington, and *S*. Muenchen. ESBL production is an important mechanism of resistance for enterobacteria. Further analysis of the putative positive isolates in the current study confirmed that 27.01% (77/285 analysis) of all ESBL tests realized with the serotypes produced a profile of resistance consistent with β-lactamase production. All tests for *S*. Minnesota serotype had ESBL phenotype. Regarding to antibiotics, aztreonam resistance was the least common amongst the *Salmonella* isolates (10%), followed by ceftazidime (14%), ceftriaxone (21%), cefoxitin (23%), and cefotaxime (32%) (Figure 2). Moreover, a difference of ≥4 mm between the radius of the inhibition zone and the radius of the truncated zone (cefoxitin (inducer) and β-lactam chosen, ceftazidime) indicated the presence of inducible chromosomal ESBL in 14 different isolates of the serotypes analyzed (Table 1 and Table 2).

**Table 1:** Average inhibition zone diameters (mm) for the tested antibiotics for each of the *Salmonella* serotypes in three analysis.

*Salmonella enterica* subsp. *enterica* (Serotypes)	ATM	CAZ	FOX	CRO	CTX
S. Linvingstone	31	25	25	29	31
S. Virchow	30	26	25	30	30
S. Mbandaka	30	22	24	30	28
S. Albany	33	27	27	33	32
S. Carrau	30	23	26	27	30
S. Shwarzenground	28	26	26	27	27
S. Minnesota	30	25	25	24	26
S. Oranienburg	32	25	25	30	32
S. Meleagridis	30	26	25	28	30
S. Worthington	28	26	25	29	27
S. Muenchen	28	24	24	27	24
S. Tennessee	30	26	26	27	28
S. Enteritidis	0	7	27	24	19
S. Mandelia	10	11	25	21	18
S. Hadar	0	10	19	16	17
S. Anatum	33	26	27	27	29
S. Agona	31	25	25	27	28
S. Cubana	21	21	24	29	28
S. Rissen	29	26	22	28	28

ATM, aztreonam; CAZ, ceftazidime; FOX, cefoxitin; CRO, ceftriaxone; CTX, cefotaxime (30 μg each).

**Table 2:** Results for extended spectrum β-lactamase-producing and AmpC β-lactamase-producing *Salmonella enterica* subsp. *enterica* (Serotypes) after three analysis.

Serotypes	ESBL			
	1° test	2° test	3° test	AmpC
*S.* Linvingstone	−	−	+	−
*S*. Virchow	+	+	−	+
*S*. Mbandaka	+	+	+	+
*S*. Albany *	−	−	−	−
*S*. Carrau	+	+	−	+
*S*. Schwarzenground	+	+	+	+
*S*. Minnesota	+	+	+	+
*S*. Oranienburg	−	+	−	+
*S*. Meleagridis *	−	−	−	−
*S*. Worthington	+	+	+	+
*S*. Muenchen	+	+	+	+
*S*. Tennessee	−	+	−	−
*S*. Enteritidis	+	+	+	+
*S*. Mandelia	+	+	+	+
*S*. Hadar	+	+	+	+
*S*. Anatum	−	−	+	+
*S*. Agona	−	+	−	−
*S*. Cubana	+	+	+	+
*S*. Rissen	+	+	+	+

* No profile of ESBL production.

The number of *Salmonella* serotypes showing ESBL production in the current study was similar with previous studies, which showed levels of ESBL production of 30.0%–60.0% amongst tested Enterobacteriaceae [15,16]. Ten serotypes were positive for ESBL in all tests, and only *S.* Albany and *S*. Meleagridis were ESBL negative in all of them. A lack of standardization of the distance between the disks is the main difficulty of the disk diffusion method. Paterson and Bonomo [15] reviewed the double diffusion test against genotypically-confirmed ESBL producers and non-producers and showed sensitivities from 79.0% to 97.0% and specificities ranging from 94.0% to 100% 

The production of β-lactamase enzymes is an important mechanism of resistance to β-lactam antibiotics. These enzymes work by hydrolyzing the β-lactam ring by breaking the amide bond. This means that the antibiotic can no longer inhibit the synthesis of the bacterial cell wall [11,12,17]. ESBL-producing strains are most commonly identified in hospitals where antibiotic use is frequent and the patient’s condition is critical [18]. However, in this present study, ESBL-producing *Salmonella* isolates were recovered from a broiler production chain. This has significant implications for public health, as the killing and processing practices can contribute to the spread of microorganisms from a carcass to other chicken meat products, introducing pathogens into the human food chain [19]. Even a small number of infected chickens may cause contamination of an entire slaughter line, multiplying the possibility of an outbreak of food poisoning in the human population [20,21]. 

In past, the widespread use of β-lactam antibiotics to treat *Salmonella* infections in both animals and humans has contributed to the rise of antibiotic-resistant microorganisms in both veterinary and human medicine [22,23]. Even despite the prohibition of drugs as additives in animal feed, extensive use of antibiotics in poultry farming was common as a result of the emergence of generics for use in food and water that are much cheaper than the primary products [24]. In Brazil, Ordinance No. 193, 1998 of the Ministry of Agriculture, Livestock and Supply, revoked by IN 9/07/2009 [25,26] which prohibits the use of chloramphenicol, penicillins, tetracyclines and sulfonamides was crucial to the reduction of these agents for veterinary use, and as growth promoters in animal feed. Although Névoa *et al.* [27] cite studies indicating that the removal of these additives in animal feed has impacted on the performance of broiler chickens, the elimination of these agents is becoming uniform throughout the world.

Data from the SENTRY Antimicrobial Surveillance Program indicated that incidences of ESBL-producing Enterobacteriaceae in Latin America, including Brazil, are among the highest in the world. In addition, ESBL-producing strains with resistance to cephalosporins, penicillins, and monobactams also show high rates of resistance to most other antimicrobial drugs [28,29]. The introduction of oxyimino-β-lactams was driven by the emergence of new β-lactamases. Some of these new β-lactamase derivatives, such as TEM and VHS, result from mutations in the genes of existing β-lactamases, which lead to a change in the profile of the substrate. 

Other new β-lactamases, such as those derived from CTX-M, are derived from chromosomal β-lactamases that are naturally-occurring in other enterobacterial species [2,9]. Thus, susceptibility profiling and other methods for identifying strains are important tools in determining the auxiliary source of infection, as well as links between human and food-animal strains [30].

## 4. Conclusions 

ESBL-producing bacteria are no longer restricted to hospitals, and are widespread throughout the environment. In this study, we reinforce the importance of ESBL-producing enterobacteria as aggravating agents of multidrug resistance in antimicrobial therapy. Some of the *Salmonella* serotypes examined in this study showed resistance to multiple β-lactam antibiotics, increasing the need for the implementation of control measures and rational use of antimicrobials to prevent, or at least reduce, the spread of antimicrobial resistance. Further research is needed to elucidate the molecular mechanisms of resistance to better understand the epidemiology of the species of ESBL-producing enterobacteria.
